# A high-resolution genetic linkage map and QTL fine mapping for growth-related traits and sex in the Yangtze River common carp (*Cyprinus carpio haematopterus*)

**DOI:** 10.1186/s12864-018-4613-1

**Published:** 2018-04-02

**Authors:** Xiu Feng, Xiaomu Yu, Beide Fu, Xinhua Wang, Haiyang Liu, Meixia Pang, Jingou Tong

**Affiliations:** State Key Laboratory of Freshwater Ecology and Biotechnology, Institute of Hydrobiology, Chinese Academy of Sciences, Wuhan, 430072 China

**Keywords:** Genetic map, QTL mapping, Growth-related traits, Sex, Candidate gene, *Cyprinus carpio Haematopterus*

## Abstract

**Background:**

A high-density genetic linkage map is essential for QTL fine mapping, comparative genome analysis, identification of candidate genes and marker-assisted selection for economic traits in aquaculture species. The Yangtze River common carp (*Cyprinus carpio haematopterus*) is one of the most important aquacultured strains in China. However, quite limited genetics and genomics resources have been developed for genetic improvement of economic traits in such strain.

**Results:**

A high-resolution genetic linkage map was constructed by using 7820 2b-RAD (2b-restriction site-associated DNA) and 295 microsatellite markers in a F2 family of the Yangtze River common carp (*C. c. haematopterus*). The length of the map was 4586.56 cM with an average marker interval of 0.57 cM. Comparative genome mapping revealed that a high proportion (70%) of markers with disagreed chromosome location was observed between *C. c. haematopterus* and another common carp strain (subspecies) *C. c. carpio*. A clear 2:1 relationship was observed between *C. c. haematopterus* linkage groups (LGs) and zebrafish (*Danio rerio*) chromosomes. Based on the genetic map, 21 QTLs for growth-related traits were detected on 12 LGs, and contributed values of phenotypic variance explained (PVE) ranging from 16.3 to 38.6%, with LOD scores ranging from 4.02 to 11.13. A genome-wide significant QTL (LOD = 10.83) and three chromosome-wide significant QTLs (mean LOD = 4.84) for sex were mapped on LG50 and LG24, respectively. A 1.4 cM confidence interval of QTL for all growth-related traits showed conserved synteny with a 2.06 M segment on chromosome 14 of *D. rerio*. Five potential candidate genes were identified by blast search in this genomic region, including a well-studied multi-functional growth related gene, *Apelin*.

**Conclusions:**

We mapped a set of suggestive and significant QTLs for growth-related traits and sex based on a high-density genetic linkage map using SNP and microsatellite markers for Yangtze River common carp. Several candidate growth genes were also identified from the QTL regions by comparative mapping. This genetic map would provide a basis for genome assembly and comparative genomics studies, and those QTL-derived candidate genes and genetic markers are useful genomic resources for marker-assisted selection (MAS) of growth-related traits in the Yangtze River common carp.

**Electronic supplementary material:**

The online version of this article (10.1186/s12864-018-4613-1) contains supplementary material, which is available to authorized users.

## Background

A genetic linkage map is an essential tool in many genetics and genomics researches, such as quantitative trait loci (QTL) mapping for target traits of economic importance [1], positional or candidate gene cloning [2], construction of gene-centromere maps [3], comparative genome analysis [4] and genome assembly [5]. In most important aquaculture species, genetic maps have been constructed using amplified fragment length polymorphism (AFLP) and microsatellite (SSR) markers [6]. However, most of these maps have few molecular markers and low density owing to the high cost and laborious wet lab work of marker discovery and genotyping, which limits their abilities to perform fine-scale QTL mapping and other studies. With the development of the next-generation sequencing technologies (NGS), a variety of genotyping-by-sequencing (GBS) methods have been developed and applied for rapidly and cost-effectively developing and genotyping thousands of single nucleotide polymorphism (SNP) markers which are available for constructing high-resolution linkage maps [7]. Among those available GBS techniques, restriction site-associated DNA (RAD) sequencing [8] and its derivative methods, such as ddRAD [9], GGRS [10], SLAF [11] and 2b-RAD [12], are reduced representation approaches that construct sequencing libraries from a fraction of the genome produced by using restriction enzymes. RAD-related sequencing technologies have been successfully used for constructing high-resolution linkage maps in several aquaculture species, such as Atlantic salmon (*Salmo salar*) [13], Japanese flounder (*Paralichthys olivaceus*) [14], tilapia (*Oreochromis niloticus* L.) [15], Zhikong Scallop (*Chlamys farreri*) [5], pearl oyster (*Pinctada fucata martensii*) [16], Chinese mitten crab (*Eriocheir sinensis*) [17] and Asian seabass (*Lates calcarifer*) [18]. Among those RAD-related methods, 2b-RAD strategy may have the simplest protocol for library preparation without any extra procedures for fragment selection and purification, and has been used for map construction in aquaculture species [5, 19, 20]. Furthermore, 2b-RAD produces uniform fragments for sequencing, thus providing more effective utilization of sequencing data among all individuals investigated.

Growth, one of the most important economic traits for aquaculture fish, is a quantitative trait that controlled by multi-gene QTLs across the genome and environmental effects. Traditional strategies of genetic improvement for growth-related traits have mainly relied on family and individual selection based on phenotype and pedigree information [21]. Nowadays, marker-assisted selection (MAS) using markers linked to QTLs has become a valuable tool to improve the accuracy of selection and speed up the genetic improvement [22]. QTL mapping enables us not only to detect genetic markers associated with the genetic variation for important traits but also to identify the candidate genes involving physiological processes of the traits [23], showing its strong application potential in MAS program. QTL analyses of growth-related traits have been conducted in some aquaculture fishes, such as Atlantic salmon [24], rainbow trout (*Oncorhynchus mykiss*) [25], tilapia [26], Asian seabass [18], Japanese flounder [27] and half-smooth tongue sole (*Cynoglossus semilaevis*) [28]. In most cases, growth-related QTLs are mapped to several linkage groups (LGs), among them few QTLs are identified on a genome-wide scale and others on chromosome-wide significance levels. Significant QTLs for sex determination, disease resistance and anti-stress traits have also been investigated for fish species [29].

Common carp (*Cyprinus carpio*), one of the most important cyprinid species, is mainly cultured in Europe and Asia with a culture history of several thousand years, and contributes an annual production of 4.1 million metric tons in the world (FAO, 2013). Based on morphological characters, common carp was classified into three subspecies: *C. c. carpio*, *C. c. haematopterus* and *C. c. rubrofuscus* [30]. Owing to the important roles of common carp in aquaculture industry and biological studies, various genetic and genomic resources, such as genetic linkage maps [31], transcriptome [32], microRNA [33] and genome sequence [34] have been developed in the Songpu mirror carp strain which belongs to the subspecies *C. c. carpio*. The Yangtze River common carp, belonging to the subspecies *C. c. haematopterus* and as one of the most important aquacultured strains in China, has quite limited genetics and genomics resources for analysis of economic traits so far. The analysis of sequence variations obtained from genome resequencing has revealed that *C. c. carpio* and *C. c. haematopterus* are grouped into the European and Asian clades respectively [34]. Recently, a high-resolution genetic map has been constructed for another strain of *C. c. haematopterus*, Yellow River carp, revealing a puzzling finding that a high proportion (62.3%) of markers with disagreed chromosome location was observed between *C. c. carpio* and *C. c. haematopterus* [35]. Construction of the high-resolution map for the Yangtze River strain of common carp would provide more information to understand the genome structure and gene rearrangement between the two subspecies. QTLs for growth-related traits have been identified by several studies in the Songpu mirror carp strain mainly based on microsatellite markers [36–38]. Compared with SNPs, microsatellites have longer flanking DNA sequences which are effective for comparative mapping. However, the density of the map base on microsatellites is generally medium or low, which is not suitable for fine mapping of QTL.

The main objectives of this study include: (1) construction of a high-resolution genetic linkage map based on 2b-RAD markers and microsatellite markers in the Yangtze River common carp *C. c. haematopterus*, (2) comparative genome analysis between the genetic map and the assembled genomes of *C. c. carpio*, zebrafish (*Danio rerio*) and grass carp (*Ctenopharyngodon idellus*), (3) performing fine QTL mapping for growth-related traits and sex, and (4) identification of candidate genes associated with growth-related traits.

## Results

### Characteristics of the phenotypic traits

The mapping family in this study consists of 104 *C. c. haematopterus* progeny, and the phenotypic growth-related traits were all in concordance with normal distribution (*P* < 0.001 for all). The average values of body weight (BW), total length (TL), body length (BL), body height (BH) and head length (HL) were 369.9 ± 89.2 g, 27.4 ± 2.5 cm, 24.9 ± 2.4 cm, 7.9 ± 0.7 cm and 6.7 ± 0.6 cm, respectively. These growth-related traits showed a strong correlation with each other (*r* = 0.780–0.993, *P* < 0.001 for all) (Table 1). The highest correlation value (*r* = 0.993) was observed between TL and BL. The BW strongly correlated with TL (*r* = 0.934), BL (r = 0.934) and BH (*r* = 0.931). By phenotype sexing in the mapping family, 50 and 54 individuals were identified as males and females, respectively, with a sex ratio of 1:1.08.

**Table 1:** Pearson correlation coefficients (r) for all pairwise combinations of the five growth-related traits (*P* < 0.001 for all)

Traits	TL	BL	BH	HL	BW
TL	1				
BL	0.993	1			
BH	0.849	0.841	1		
HL	0.782	0.782	0.723	1	
BW	0.934	0.934	0.931	0.780	1

*TL* total length, *BL* body length, *BH* body height, *HL* head length, *BW* body weight

### 2b-RAD and microsatellite genotyping

Nearly 0.18 million potential *Bcg*I restriction sites were estimated from the assembled genome sequence of common carp [34]. By single-end sequencing, a total of 263.98 million reads were generated from 2b-RAD libraries, including 7.61 million reads from the female parent, 10.22 million reads from the male parent and 246.15 million reads from the progenies (2.37 million reads per progeny). After quality filtering and sequence trimming, parental reads were clustered into 121,494 representative tags, including 91,591 parent-shared tags (codominant tags) and 29,903 parent-specific tags (dominant tags). After filtering low-quality tags with low (< 8) or high (> 3000) coverage, 83,924 codominant tags and 24,075 dominant tags were remained and used for constructing reference tags. The reads of offspring were mapped on the reference tags after quality filtering and sequence trimming. Finally, a total of 19,839 polymorphic markers including 12,084 codominant (parent-shared) markers and 7755 dominant (parent-specific) markers were heterozygous in at least one parent and genotyped in at least 80% of the offspring.

Among the 1500 microsatellites, 416 were reliably amplified polymorphic products in the mapping family. In total, 20,255 markers including above 2b-RAD and SSR markers were remained, and were tested for their segregation distortion. The results showed that 8921 markers including 3748 2b-RAD codominant markers, 4830 2b-RAD dominant markers and 343 SSR markers were in accordance with the Mendelian expectations (*P* ≥ 0.05) and were used for linkage analysis. These markers showed five segregation types: <ab × cd > (*n* = 14), <ef × eg > (*n* = 28), <hk × hk > (*n* = 373), <lm × ll > (*n* = 4971) and < nn × np > (*n* = 3535).

### Construction of the high-resolution linkage map

**Fig. 1:**
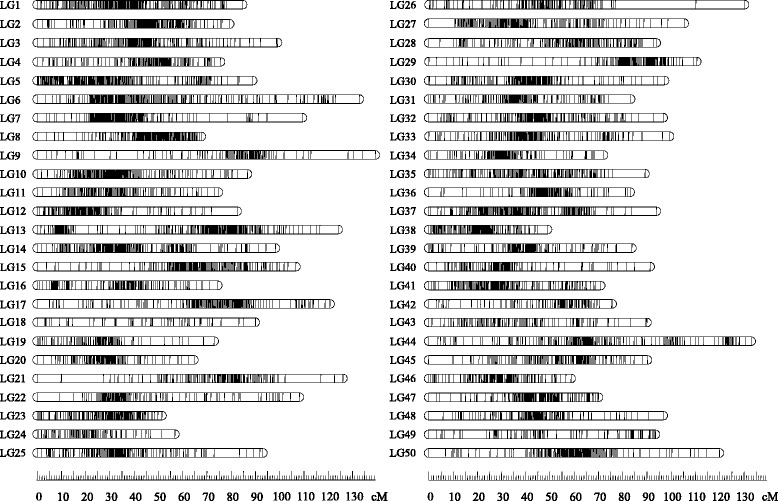
The sex-averaged genetic linkage map of the Yangtze River common carp *C. c. haematopterus* constructed based on 2b-RAD and microsatellite markers

By using the JoinMap 4.1 software [39] with the logarithm of odds (LOD) threshold of 11.0, 8115 markers (7820 2b-RAD markers and 295 SSRs) (Additional file 1: Table S1) were successfully grouped into 50 linkage groups (LGs) which was consistent with the haploid chromosome number of common carp [38]. The sex-averaged genetic map spanned 4586.56 cM with an average marker interval of 0.57 cM. The genetic length of LGs ranged from 49.05 cM (LG38) to 139.52 cM (LG9) with an average of 91.73 cM, and the number of markers varied from 89 (LG24) to 262 (LG13) with an average of 162 (Fig. 1 and Table 2). Using two different methods [40, 41], the genome length was estimated to be 4587.70 cM (G_e1_) and 4645.93 cM (G_e1_), with an average of 4616.82 cM which was used as the expected genome length. Therefore, the genome coverage (C_of_) of this genetic map was 99.3%. Since the genome size of common carp has been estimated to be 1.70 Gb based on cytogenetic method [42], the average recombination rate across all LGs was ~ 2.7 cM/Mb.

**Table 2:** Summary of the sex-averaged genetic linkage map of *C. c. haematopterus*

Linkage group	Number of markers	Genetic length (cM)	Marker interval (cM)	Linkage group	Number of markers	Genetic length (cM)	Marker interval (cM)
1	230	84.81	0.37	26	117	130.08	1.11
2	202	79.33	0.39	27	194	105.41	0.54
3	191	99.11	0.52	28	167	93.80	0.56
4	164	75.62	0.46	29	155	110.47	0.71
5	238	88.88	0.37	30	172	97.30	0.57
6	253	132.94	0.53	31	140	83.34	0.60
7	235	109.44	0.47	32	177	96.74	0.55
8	208	67.66	0.33	33	182	99.28	0.55
9	120	139.52	1.16	34	151	72.11	0.48
10	203	86.65	0.43	35	185	89.15	0.48
11	144	74.81	0.52	36	145	83.09	0.57
12	157	82.47	0.53	37	196	93.89	0.48
13	262	124.18	0.47	38	131	49.05	0.37
14	214	98.26	0.46	39	142	83.78	0.59
15	183	106.71	0.58	40	121	91.32	0.75
16	145	74.49	0.51	41	171	70.94	0.41
17	184	120.83	0.66	42	108	75.74	0.70
18	132	90.00	0.68	43	106	89.97	0.85
19	102	73.04	0.72	44	188	133.00	0.71
20	120	64.74	0.54	45	140	90.28	0.64
21	133	126.07	0.95	46	129	58.67	0.45
22	127	108.16	0.85	47	133	69.99	0.53
23	151	51.55	0.34	48	138	96.70	0.70
24	89	56.83	0.64	49	123	93.48	0.76
25	146	93.05	0.64	50	171	119.86	0.70
				Total	8115	4586.56	0.57

### Comparative genome mapping

**Fig. 2:**
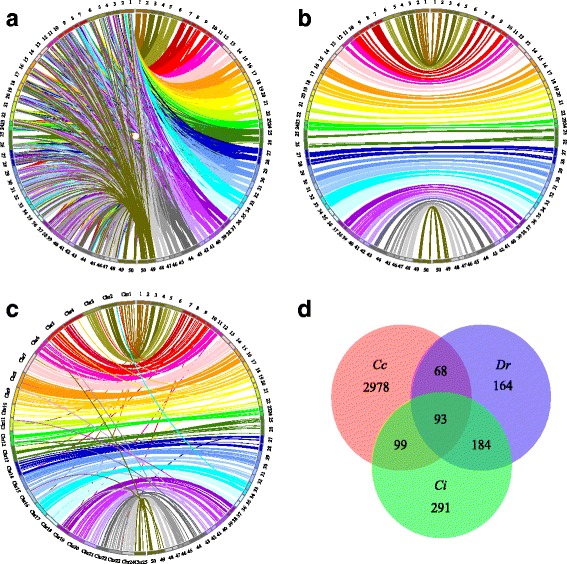
Circos diagram representing syntenic relationships between *C. c. haematopterus* (right) and (**a** and **b**) *C. c. carpio* (left) and (**c**) *Danio rerio* (left), and (**d**) Venn diagrams describing overlaps among uniquely aligned markers that mapped to genomes of *C. c. carpio* (*Cc*), *D. rerio* (*Dr*) and *C. idellus* (*Ci*). Only markers on each linkage group of *C. c. haematopterus* that were mapped to a single linkage group of *C. c. carpio* were shown in (b)

Through blast search, a total of 6002 (74.0%) markers were aligned to the assembled genome of *C. c. carpio*, among them, 3238 (53.9%) were uniquely mapped on the assembled LGs and were used for synteny analysis. Overall, a one-to-one correspondence was observed between LGs of *C. c. haematopterus* and *C. c. carpio*. However, for each LG of *C. c. haematopterus*, about an average proportion of 30% of markers were located on a single LG of *C. c. carpio*, others were dispersed on various LGs (Fig. 2a, Fig. 2b and Additional file 2: Figure S1).

A total of 509 markers on linkage map of *C. c. haematopterus* were uniquely aligned to the chromosomes of *D. rerio* (Fig. 2c and Additional file 3: Figure S2), with 475 (93.3%) markers located into 50 syntenic boxes. Every two LGs of *C. c. haematopterus* were homologous with a single chromosome of *D. rerio*, showing a clear 2:1 relationship of *C. c. haematopterus* LGs and *D. rerio* chromosomes. A high level of genomic synteny was also detected between *C. c. haematopterus* and *C. idellus* (Additional file 4: Figure S3). Of the 667 markers uniquely anchored to the assembled LGs of *C. idellus*, 622 (93.3%) were located into syntenic boxes. Four LGs (LG19, LG20, LG43 and LG44) of *C. c. haematopterus* linkage map were mapped to LG24 of *C. idellus*, while a clear 2:1 syntenic relationship was observed between the remaining 46 LGs of *C. c. haematopterus* and 23 LGs of *C. idellus*. Among all aligned markers, 93 (55 2b-RAD markers and 38 SSRs) were uniquely aligned to all reference genomes (Fig. 2d), revealing that these markers were highly conserved among the three cyprinid fishes.

### Fine QTL mapping for growth-related traits and sex

**Fig. 3:**
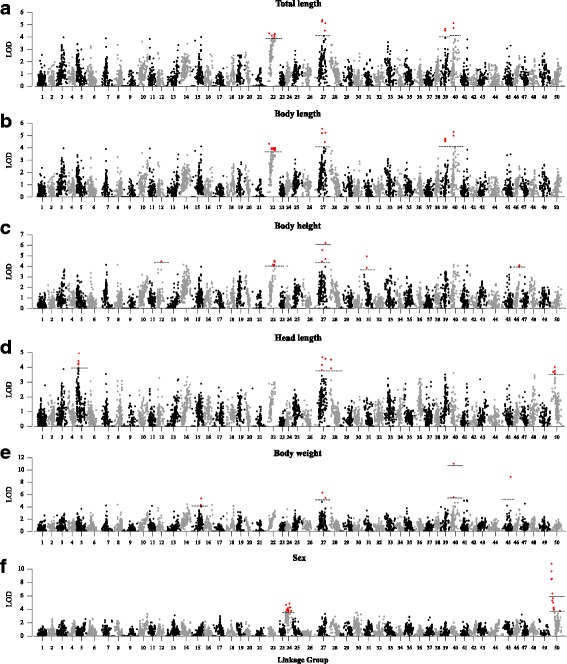
A genome scan of LOD profiles for (**a**) total length, (**b**) body length, (**c**) body height, (**d**) head length, (**e**) body weight and (**f**) sex in *C. c. haematopterus*. The dashed and solid lines indicated the chromosome-wide and genome-wide significance thresholds

The chromosome-wide and genome-wide LOD significance thresholds for growth-related traits varied from 3.3 to 6.2 and 6.1 to 10.2, respectively, based on permutation test. By using multiple QTL model (MQM), twenty one QTLs associated with growth-related traits were detected on 12 LGs, including two genome-wide significant QTLs (qBH27-a and qBW40-a) and 19 chromosome-wide significant QTLs, with LOD scores ranging from 4.02 to 11.13 (Fig. 3 and Table 3). Four QTLs (qTL22-a, qTL27-a, qTL39-a and qTL40-a) associated with TL were located at 68.57 cM, 70.23 cM, 41.29 cM and 29.36 cM along LG 22, LG 27, LG 39 and LG40, and contributed values of phenotypic variance explained (PVE) of 18.3, 20.3, 18.6 and 20.3%, respectively (Fig. 3a). Owing to the high correlation value (*r* = 0.993) between TL and BL, QTLs for BL were located at the same confidence intervals along four LGs (Fig. 3b). Five QTLs for BH were mapped to LG12, LG22, LG27, LG31 and LG46 with values of PVE of 18.0, 18.2, 24.2, 19.7 and 16.7%, respectively (Fig. 3c). Four QTLs associated with HL were located on four LGs (LG5, LG27, LG28 and LG50) with values of PVE ranging from 16.3 to 18.4% (Fig. 3d). For BW, four QTLs located on LG15, LG27, LG40 and LG45 contributed values of PVE of 21.2, 21.7, 38.6 and 32.5%, with LOD scores of 5.38, 5.51, 11.13 and 8.89, respectively (Fig. 3e). Among all confidence intervals of QTLs, only the confidence interval on LG27 was associated with all five growth-related traits.

**Table 3:** Detected QTLs associated with five growth traits in *C. c. haematopterus*

Trait	QTL	LG	CI (cM)	Nearest marker	LOD	LOD threshold		PVE (%)
						GW	CW	
Total length	qTL22-a	22	64.71–72.73	ref-88,098	4.57	6.3	3.9	18.3
	qTL27-a	27	69.23–70.62	ref-90,952	5.13		4.1	20.3
	qTL39-a	39	41.26–41.34	ref-109,024	4.66		4	18.6
	qTL40-a	40	29.09–29.97	ref-13,303	5.13		4.1	20.3
Body length	qBL22-a	22	64.71–72.73	ref-88,098	4.65	6.5	3.7	18.6
	qBL27-a	27	69.23–70.62	ref-90,952	5.27		4.1	20.8
	qBL39-a	39	41.26–41.34	ref-109,024	4.75		4.1	19
	qBL40-a	40	29.09–29.97	ref-13,303	5.29		4.1	20.9
Body height	qBH12-a	12	39.47–42.79	ref-107,053	4.48	6.1	4.3	18
	qBH22-a	22	58.84–69.59	ref-35249_8	4.54		4.1	18.2
	qBH27-a	27	69.23–70.62	ref-90,952	6.27		4.3	24.2
	qBH31-a	31	23.19–23.65	ref-89691_6	4.96		3.8	19.7
	qBH46-a	46	50.83–52.57	ref-37003_10	4.11		3.9	16.7
Head length	qHL5-a	5	16.53–18.39	ref-44,945	4.43	5.6	3.8	17.8
	qHL27-a	27	69.23–71.09	ref-90,952	4.6		3.8	18.4
	qHL28-a	28	13.31–14.66	ref-97,775	4.53		3.8	18.2
	qHL50-a	50	40.12–44.71	ref-94,980	4.02		3.6	16.3
Body weight	qBW15-a	15	78.61–83.20	ref-43,553	5.38	10.2	4.6	21.2
	qBW27-a	27	69.23–70.62	ref-90,952	5.51		5.1	21.7
	qBW40-a	40	29.09–29.97	ref-58277_27	11.13		6.2	38.6
	qBW45-a	45	64.91–65.76	ref-88407_25	8.89		5	32.5

*LG* Linkage group, *CI* Confidence interval, *GW* Genome-wide, *CW* chromosome-wide, *PVE* Phenotypic variance explained

For sex, the genome-wide LOD significance threshold was 5.9. A genome-wide significant QTL was finely mapped on LG50 with the confidence interval ranging from 12.25 cM to 40.75 cM, and explained 38.1% of the phenotypic variance (LOD = 10.83) (Fig. 3f). A total of 15 markers were identified in this QTL region, among them, nine continuous markers (from ref-20,177 to ref-119,060) with the average LOD score of 7.12 were all heterozygous in female parent and homozygous in male parent. The chromosome-wide LOD significance thresholds for sex varied from 3.2 (LG21) to 3.9 (LG32). Three chromosome-wide significant QTLs were detected on LG24, and contributed value of PVE of 18.7%, 16.9 and 22.2%, with LOD scores of 4.67, 4.19 and 5.66, respectively.

### Potential candidate genes for growth

**Fig. 4:**
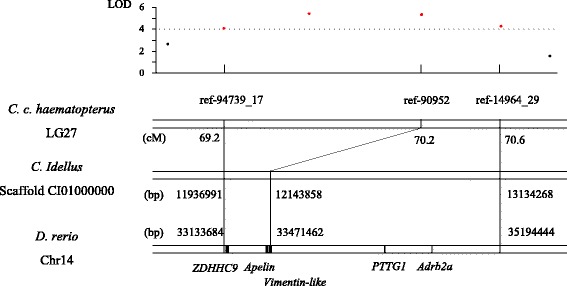
The QTL region for growth traits on LG27 of *C. c. haematopterus* and its homologous region in genomes of *Danio rerio* and *Ctenopharyngodon idellus*

By searching against the non-redundant (nr) protein database, the sequences of six QTL markers for growth-related traits showed high similarities to fish genes (Additional file 5: Table S2). On the other hand, the homologous regions of QTLs were identified in the assembled genomes of *D. rerio* and *C. idellus*. On LG27 of *C. c. haematopterus*, a confidence interval of QTL for all growth-related traits ranging from 69.2 cM to 70.6 cM was mapped to a 2.06 M region on chromosome 14 of *D. rerio* and a 1.20 M region on scaffold CI01000000 of *C. idellus* (Fig. 4). Based on the annotation information of *D. rerio* genome, 36 genes were located in this region. Among them, four genes, *ZDHHC9* (zinc finger, DHHC-type containing 9), *Apelin*, *PTTG1* (pituitary tumor-transforming 1) and *Adrb2a* (adrenoceptor beta 2, surface a) have been reported to play important roles in growth of skeletal muscle [43], obesity [44], tumorigenesis [45] and growth of muscle myotomal fibres [46], respectively, and a vimentin-like gene contains the sequence of marker ref-90,952. The confidence interval of the QTL (qHL5-a) on LG5 was mapped to a 0.87 M region on chromosome 3 of *D. rerio* and a 0.63 M region on Scaffold CI01000034 of *C. idellus*. However, this QTL region was observed inversion compared to genome sequence of *D. rerio* and *C. idellus* (Additional file 6: Figure S4), indicating that the rearrangement of chromosome might occur in this region between *C. c. haematopterus* and *D. rerio* and *C. idellus*. According to the annotation of *D. rerio* genome, two potential growth-related genes, *Atf4b2* (activating transcription factor 4b2) and *Pvalb6* (parvalbumin 6) were located in this region (Additional file 6: Figure S4). These candidate QTL genes may involve in the genetic control of growth-related traits, which are worthy of further studies.

## Discussion

In this study, 2b-RAD sequencing of a F2 family of common carp generated 8578 high-quality markers which were more than that obtained by RAD sequencing [34]. However, only 4% of these markers were polymorphic in both parents, which is similar to that reported in the sea cucumber [47]. Additional microsatellite markers were genotyped in this study, and 14% of them were parent-shared markers which help us to construct the first high-density sex-averaged genetic map of the Yangtze River common carp (*C. c. haematopterus*). The number of LGs (*n* = 50) (Fig. 1) is in agreement with the haploid chromosome number of common carp [48]. Compared with the genetic map of Songpu mirror carp (*C. c. carpio*) [34], this map had a slightly larger size (4586.56 vs 3946.7 cM) and a higher resolution (0.57 vs 0.93 cM). On LG21 and LG26, few markers were located at the terminal region, which may be caused by the presence of centromeres [3], and/or the effect of DNA methylation. Attention should be paid to the caution of mapped markers at the terminal region and the effect of DNA methylation as this might involve in the reality and accuracy of some SNP loci. However, it is unclear whether *Bcg*I (type IIB endonuclease) used in the 2b-RAD sequencing of this study is sensitive to methylation or not [49–51], currently we have difficulties in dressing this issue clearly. In future, we should firstly clarify the sensitivity of *Bcg*I to methylation and then investigate the relationship between the caution of mapped markers at the terminal region and the effect of DNA methylation.

With a long history of cultivation, common carp has been bred into numerous strains and local populations which are mainly grouped into European and Asian clades based on sequence variations [34]. In this study, the syntenic analysis was performed between the high-density genetic map of *C. c. haematopterus* (Asian) and the assembled genome of *C. c. carpio* (European) to investigate their evolutionary relationship. The results showed that a 1:1 relationship between LGs of *C. c. haematopterus* and *C. c. carpio* was observed based on syntenic boxes, however, only 30% of markers were located in these syntenic boxes (Additional file 2: Figure S1), which was similar to that observed from the strain of Yellow River carp *C. c. haematopterus* [35], indicating the extensive intra-chromosomal rearrangements between the two subspecies. Common carp is believed to have undergone a fourth round of genome duplication [34, 52], which was also validated in this study. A high level of conserved genomic synteny was observed between *C. c. haematopterus* and *D. rerio* (93.3%) with a clear 2:1 relationship between LGs and chromosomes (Fig. 2c and Additional file 3: Figure S2). Similarly, a clear 2:1 relationship was also observed between LGs of *C. c. haematopterus* and *C. idellus* except for LG24 of *C. idellus*, which showed synteny with LG19, LG20, LG43 and LG44 of *C. c. haematopterus* (Additional file 4: Figure S3). Therefore, two chromosomes (Chr10 and Chr22) of *D. rerio* were homologous with LG24 of *C. idellus*, which has also been reported in previous studies [53, 54]. According to the hypothesis that the ancestor of teleosts had 24 chromosomes [55, 56], the two chromosomes (Chr10 and Chr22) of *D. rerio* may be formed by the fission of an ancestral chromosome.

Strong correlations among the five growth-related traits (BW, TL, BL, BH and HL) have been reported in some aquaculture species, such as Atlantic salmon [57], blunt snout bream (*Megalobrama amblycephala*) [58] and Zhikong Scallop [5]. Similarly in this study, five growth-related traits were highly correlated with each other, with the highest correlation value (*r* = 0.99) between TL and BL and relatively low correlation value (mean *r* = 0.76) between HL and other four traits. As expected, QTLs for TL and BL had the same distribution patterns on four LGs, while three of four QTLs for HL were located on three LGs which contain no QTL for other growth-related traits (Table 3). On LG27, a QTL-containing interval was identified for all growth-related traits, suggesting the influence of one QTL on all growth-related traits in the Yangtze River carp. QTLs for growth on LG27 have also been identified in Yellow River carp [36], further comparative analysis should be performed to verify the common loci that have genetic effects on growth in both strains of *C. c. haematopterus*.

The high-density genetic map used for QTL analysis in this study had a higher resolution than previous used maps [36–38]. The 1.4 cM confidence interval of QTL for all growth traits showed synteny with a 2.06 M region on chromosome 14 of *D. rerio* which contains 36 genes (Fig. 4). Another 0.8 cM confidence interval of QTL for HL was mapped to a 0.87 M region on chromosome 3 of *D. rerio* containing 23 genes (Additional file 6: Figure S4). Potential candidate genes were identified based on previous study of gene function. Among them, two genes, *Apelin* and *Pvalb6* are the most likely to be associated with growth. Apelin, an adipokine, is expressed and secreted by adipocytes, and has been reported to have significant effects on energy metabolism, insulin sensitivity and pituitary hormone release [45, 59, 60]. Parvalbumins are extremely abundant in fish muscle and play an important role in muscle relaxation [61]. A microsatellite polymorphism in *Pvalb1* has been reported to be significantly associated with body weight and body length in Asian seabass [61]. Further studies are necessary to verify the associations between polymorphisms in these two genes with growth traits in common carp.

The sex determination of common carp is male-dominant (XX/XY) since sex ratios in conventional diploid offspring approximate 1:1 and gynogenetic offspring are all female [62]. However, like other fish species, it is difficult to distinguish between the sex chromosomes (X and Y), as well as between the sex and autosomal chromosomes based on current cytogenetic techniques [63, 64]. In this study, QTLs for sex determination of Yangtze River carp were identified on LG24 and LG50, which was not consistent with that observed in Yellow River carp with the QTLs on LG11and LG43 [35]. Chen et al. [65] identified a sex-specific DNA marker in Yellow River carp with the marker sequence mapped on an unplaced genomic scaffold. An earlier discovery has provided evidence for autosomal influences on sex determination in common carp [66]. These results may indicate that sex determination of common carp is polygenic and different genes may influence sex determination in different strains. Such polygenic sex determination mechanism has also been observed in other fish species, such as zebrafish [67, 68], tilapia [15, 69] and Atlantic salmon [70]. The sex-determining loci identified in this study would be useful for MAS breeding in the Yangtze River common carp.

## Conclusions

A high-density genetic linkage map of the Yangtze River carp (*C. c. haematopterus*) was constructed based on 7820 2b-RAD markers and 295 microsatellite markers in this study. Comparative genome mapping revealed that common carp had undergone extensive intra-chromosomal rearrangements during the domestication process. A high level of syntenic relationship was observed between Yangtze River carp and zebrafish and grass carp. A total of 21 QTLs for growth-related traits were identified on 12 LGs. A set of candidate genes were identified based on the sequences of QTL-containing intervals. These genetic markers or candidate genes associated with growth traits provide a basis for marker-assisted selection (MAS) in the Yangtze River carp and/or other common carp strains.

## Methods

### Mapping family and phenotype data

Two wild populations of common carp were collected from two geographic areas (Jingzhou and Wuhan) along the Yangtze River, and were used to produce F1 populations. In late April 2011, a F2 family was generated by crossing of a dam and a sire from the the same F1 family, and was raised in a 0.3 ha muddy pond after disinfection and fertilizing at Zhangdu Lake Fish Farm (Wuhan, China). Fish were fed three times daily, with soy milk at the larval stage (about 15 days post-hatch) and pellet feed at subsequent stages. After 9 months of culture, 104 progeny used for QTL mapping were randomly selected for phenotypic measurements. Five parameters of growth-related traits including BW, TL, BL, BH and HL were recorded. The measured individuals were then PIT-tagged and continued being cultured in the pond. Phenotype sexing was performed for each progeny of the F2 family by gently press abdomen of the fish in late April 2012. In Wuhan, the time for fully maturation of common carp is one year for male and two years for females. In one year, testis is fully developed and full of sperm in spawning season (spring). If milt were observed from the genital opening, then the fish was recorded as a male, otherwise as a female (female fish also with a swollen belly, which has moderately developed or almost matured ovary inside in one year).

The distribution patterns (normal or nonnormal) of growth-related traits were determined by the Kolmogorov-Smirnov tests. To investigate the relationships among these growth-related traits, Pearson correlation coefficients were calculated using the SPSS 19.0 software (IBM, USA). Fin clips of parental and full-sib fish were sampled and ethanol-preserved. Genomic DNA was extracted from fin clips using a traditional phenol–chloroform method [71]. DNA quality was checked using 1% agarose gel electrophoresis, and the concentration and purity were inspected using NanoDrop 2000 spectrophotometer (Thermo Scientific, USA). All experimental animal programs involved in this study were approved by the Animal Care and Use Committee at the Institute of Hydrobiology, Chinese Academy of Sciences.

### 2b-RAD sequencing and de novo genotyping

Before the library preparation, potential restriction sites were calculated based on the assembled genome sequence of common carp (http://www.carpbase.org). 2b-RAD libraries were prepared for two parents and 104 progeny by following the standard protocol [12] with some modifications. 200 ng of genomic DNA from each individual was digested with *Bcg*I restriction enzyme (New England Biolabs, USA) at 37 °C for 4 h. The digestion product was heat-inactivated for 20 min at 65 °C, and then ligated to adapter 1 and adapter 2 at 16 °C over night. The ligated fragments were amplified with Phusion High-Fidelity DNA Polymerase (Thermo Scientific, USA) using a set of four primers that introduce sample-specific barcode and sequencing primers. After 15 cycles of PCR, the library was obtained by purifying the amplification products at ~ 170 bp via retrieval from 8% polyacrylamide gels. Libraries were pooled with equal amount to make the final library which was sequenced in a lane of the Illumina HiSeq2500 SE50 platform (Illumina, USA).

Raw reads were first trimmed to remove adapter sequences and the terminal 2-bp positions. Reads with no restriction sites or containing long homopolymers (more than 10 bp), ambiguous bases (N), low-quality sequences (more than 5 positions with quality of less than 20) or mitochondrial origins were removed. The remaining trimmed reads with 32 bp in length were used for subsequent analysis. De novo genotyping was performed using the RADtyping program v1.0 [72]. Using this software, both codominant and dominant markers were identified and genotyped.

### Microsatellite genotyping

A total of 1500 genomic and transcript-associated SSR markers previously published for common carp [73, 74] were also used for initial segregation screening in the mapping family. Polymorphic loci segregated in either female or male parents were genotyped in the progeny through PCR amplification. PCR was performed on a veritiTM 96 well thermal cycler (Applied Biosystems, USA) with a total volume of 12.5 μl, containing 30 ng template DNA, 1.25 μl 10× PCR buffer (TaKaRa, Japan), 0.25 U Taq DNA polymerase (TaKaRa, Japan), 50 μM each dNTP, 0.2 μM each primer and water to the final volume. The thermal cycling was programmed as follows: 5 min at 94 °C, followed by 37 cycles of 94 °C for 30 s, 35 s at appropriate annealing temperature, and 72 °C for 40 s, and the last extension at 72 °C for 10 min. PCR products were size-fractionated on 8% polyacrylamide gels and visualized by ethidium bromide staining.

### Linkage map construction

Microsatellite loci were separated into three segregation patterns: 1:1 (type lm × ll or nn × np), 1:2:1 (type hk × hk) and 1:1:1:1 (type ab × cd or ef × eg), and 2b-RAD markers just showed the first two segregation patterns. The genotyping data of 2b-RAD and microsatellite markers were integrated for further analysis. Segregating markers that could not be genotyped in at least 20% of the offspring were removed. The sex-averaged genetic linkage map was constructed using JoinMap 4.1 [39] under the CP algorithm. The “*Locus genot. Freq.*” function was used for a chi-square test to assess the goodness of fit to the expected segregation ratios for each locus at the confidence level of 0.05. Markers showing significant departure from the expected segregation ratios were excluded. Linkage between markers was examined by estimating logarithm of the odds (LOD) scores for recombination fraction. Markers were grouped at a LOD threshold score of 11.0 and a maximum recombination fraction of 0.35. The regression mapping algorithm was selected for mapping. The Kosambi mapping function was used to convert the recombination frequencies into map distances in centiMorgans (cM). MapChart 2.2 software was used for graphical visualization of the linkage groups (LGs) [75].

### Comparative genome analysis

To perform comparative analysis, sequences of the markers on the genetic map were aligned to the assembled genomes of *C. c. carpio*, *D. rerio* (GRCz10) and *C. idellus* (http://www.ncgr.ac.cn/grasscarp). For 2b-RAD markers, their sequences were mapped to genomes using the short-read alignment program Bowtie [76]. A maximum of two nucleotide mismatches and no gaps between the 2b-RAD sequence and genomes were permitted for any alignment. For SSR markers, the flanking sequences were searched against reference genomes using the basic local alignment search tool (BLAST) with the cut-off value of 1e-10. Finally, markers of which sequences were anchored to a single unique position on reference genomes were used for comparative genome mapping. The genomic synteny was visualized using the software Circos [77].

### QTL analysis of growth-related traits and sex

QTL analysis was carried out using MapQTL 6.0 program [78]. Multiple QTL model (MQM) mapping were utilized to detect any significant association between growth-related traits and marker loci in the data sets. Cofactors for MQM analyses were automatically selected with a *p*-value of 0.02. Significant LOD thresholds were calculated by permutation test of α < 0.05 and *n* = 1000 for significant linkages. Calculation of the percentage of phenotypic variance explained (PVE) by a QTL was performed on the basis of the population variance found within the progeny.

### Identification of potential candidate genes

For those markers that were located in the confidence intervals of QTLs and mapped at a single position on the assembled genome of *C. c. carpio*, their sequences were extended by adding 500 nucleotide sequences from each side in the genome, and then searched against the assembled genomes of *D. rerio* and *C. idellus* again. Two methods were used to identify potential candidate genes for growth traits. First, the extended sequences of markers were searched against the non-redundant protein sequences (nr) at the National Center for Biotechnology Information (NCBI) with a threshold of *E*-value ≤10^− 10^ using blastx to identify potential candidate genes of these markers. Second, the extended markers were used to identify conserved regions in genome of *D. rerio*, and the potential candidate genes were identified in the conserved regions based on the annotation information.

## Additional files

Additional file 1: **Table S1.** Information of markers located in the high-resolution genetic linkage map of the Yangtze River common carp (*Cyprinus carpio haematopterus*), and their aligned positions in genomes of *C. c. carpio*, *Danio rerio* and *Ctenopharyngodon idellus*. (XLSX 696 kb)

Additional file 2: **Figure S1.** Genomic synteny visualized using Oxford grids between linkage groups of *C. c. haematopterus* and LGs of *C. c. carpio*. (PDF 1100 kb)

Additional file 3: **Figure S2.** Genomic synteny visualized using Oxford grids between linkage groups of *C. c. haematopterus* and chromosomes *Danio rerio*. (PDF 32 kb)

Additional file 4: **Figure S3.** Genomic synteny visualized using Oxford grids between linkage groups of *C. c. haematopterus* and LGs of *Ctenopharyngodon idellus*. (PDF 31 kb)

Additional file 5: **Table S2.** The markers within confidence intervals of QTLs for growth traits in the Yangtze River common carp (*Cyprinus carpio haematopterus*), and their aligned positions in genomes of *C. c. carpio*, *Danio rerio* and *Ctenopharyngodon idellus*. (XLSX 15 kb)

Additional file 6: **Figure S4.** The QTL region for head length on LG5 of *C. c. haematopterus* and its homologous region in genomes of *Danio rerio* and *Ctenopharyngodon idellus*. (PDF 135 kb)

## Abbreviations

AFLP: Amplified fragment length polymorphism
LGs: Linkage groups
LOD: Logarithm of odds
MAS: Marker-assisted selection
NGS: Next-generation sequencing
PIT: Passive integrated transponders
PVE: Phenotypic variance explained
QTL: Quantitative trait loci
RAD: Restriction site-associated DNA
SNP: Single nucleotide polymorphism

## References

[1] Sun XW, Liang LQ (2004). A genetic linkage map of common carp (*Cyprinus carpio* L.) and mapping of a locus associated with cold tolerance. Aquaculture.

[2] Tamura Y, Hattori M, Yoshioka H, Yoshioka M, Takahashi A, Wu J (2014). Map-based cloning and characterization of a brown planthopper resistance gene BPH26 from *Oryza sativa* L. ssp *indica* cultivar ADR52. Sci Rep.

[3] Feng X, Wang X, Yu X, Zhang X, Lu C, Sun X (2015). Microsatellite-centromere mapping in common carp through half-tetrad analysis in diploid meiogynogenetic families. Chromosoma.

[4] Zhu C, Tong J, Yu X, Guo W (2015). Comparative mapping for bighead carp (*Aristichthys nobilis*) against model and non-model fishes provides insights into the genomic evolution of cyprinids. Mol Gen Genomics.

[5] Jiao W, Fu X, Dou J, Li H, Su H, Mao J (2014). High-resolution linkage and quantitative trait locus mapping aided by genome survey sequencing: building up an integrative genomic framework for a bivalve mollusc. DNA Res.

[6] Yue GH (2014). Recent advances of genome mapping and marker-assisted selection in aquaculture. Fish Fish.

[7] Davey JW, Hohenlohe PA, Etter PD, Boone JQ, Catchen JM, Blaxter ML (2011). Genome-wide genetic marker discovery and genotyping using next-generation sequencing. Nat Rev Genet..

[8] Baird NA, Etter PD, Atwood TS, Currey MC, Shiver AL, Lewis ZA (2008). Rapid SNP discovery and genetic mapping using sequenced RAD markers. PLoS One.

[9] Peterson BK, Weber JN, Kay EH, Fisher HS, Hoekstra HE (2012). Double digest RADseq: an inexpensive method for de novo SNP discovery and genotyping in model and non-model species. PLoS One.

[10] Liao R, Wang Z, Chen Q, Tu Y, Chen Z, Wang Q (2015). An efficient genotyping method in chicken based on genome reducing and sequencing. PLoS One.

[11] Sun X, Liu D, Zhang X, Li W, Liu H, Hong W (2013). SLAF-seq: an efficient method of large-scale de novo SNP discovery and genotyping using high-throughput sequencing. PLoS One.

[12] Wang S, Meyer E, McKay JK, Matz MV (2012). 2b-RAD: a simple and flexible method for genome-wide genotyping. Nat Methods.

[13] Gonen S, Lowe NR, Cezard T, Gharbi K, Bishop SC, Houston RD (2014). Linkage maps of the Atlantic salmon (*Salmo salar*) genome derived from RAD sequencing. BMC Genomics.

[14] Shao C, Niu Y, Rastas P, Liu Y, Xie Z, Li H (2015). Genome-wide SNP identification for the construction of a high-resolution genetic map of Japanese flounder (*Paralichthys olivaceus*): applications to QTL mapping of Vibrio anguillarum disease resistance and comparative genomic analysis. DNA Res.

[15] Palaiokostas C, Bekaert M, Khan MG, Taggart JB, Gharbi K, McAndrew BJ (2013). Mapping and validation of the major sex-determining region in Nile tilapia (*Oreochromis niloticus* L.) using RAD sequencing. PLoS One.

[16] Shi Y, Wang S, Gu Z, Lv J, Zhan X, Yu C (2014). High-density single nucleotide polymorphisms linkage and quantitative trait locus mapping of the pearl oyster, *Pinctada fucata martensii* dunker. Aquaculture.

[17] Cui Z, Hui M, Liu Y, Song C, Li X, Li Y (2015). High-density linkage mapping aided by transcriptomics documents ZW sex determination system in the Chinese mitten crab *Eriocheir sinensis*. Heredity.

[18] Wang L, Wan ZY, Bai B, Huang SQ, Chua E, Lee M (2015). Construction of a high-density linkage map and fine mapping of QTL for growth in Asian seabass. Sci Rep.

[19] Liu H, Fu B, Pang M, Feng X, Yu X, Tong J. A high-density genetic linkage map and QTL fine mapping for body weight in crucian carp (*Carassius auratus*) using 2b-RAD sequencing. G3. 2017;7:2473–87. 10.1534/g3.117.041376 PMC5555455 28600439

[20] Fu B, Liu H, Yu X, Tong J. A high-density genetic map and growth related QTL mapping in bighead carp (*Hypophthalmichthys nobilis*). Sci Rep. 2016;6(28679) 10.1038/srep28679 PMC4921863 27345016

[21] Gjedrem T (2000). Genetic improvement of cold-water fish species. Aquac Res.

[22] Sonesson A (2007). Within-family marker-assisted selection for aquaculture species. Genet Sel Evol.

[23] Mackay TF, Stone EA, Ayroles JF (2009). The genetics of quantitative traits: challenges and prospects. Nat Rev Genet.

[24] Gutierrez AP, Lubieniecki KP, Davidson EA, Lien S, Kent MP, Fukui S (2012). Genetic mapping of quantitative trait loci (QTL) for body-weight in Atlantic salmon (*Salmo salar*) using a 6.5 K SNP array. Aquaculture.

[25] Wringe BF, Devlin RH, Ferguson MM, Moghadam HK, Sakhrani D, Danzmann RG (2010). Growth-related quantitative trait loci in domestic and wild rainbow trout (*Oncorhynchus mykiss*). BMC Genet.

[26] Liu F, Sun F, Xia JH, Li J, Fu GH, Lin G (2014). A genome scan revealed significant associations of growth traits with a major QTL and GHR2 in tilapia. Sci Rep.

[27] Song W, Pang R, Niu Y, Gao F, Zhao Y, Zhang J (2012). Construction of high-density genetic linkage maps and mapping of growth-related quantitative trail loci in the Japanese flounder (*Paralichthys olivaceus*). PLoS One.

[28] Song W, Li Y, Zhao Y, Liu Y, Niu Y, Pang R (2012). Construction of a high-density microsatellite genetic linkage map and mapping of sexual and growth-related traits in half-smooth tongue sole (*Cynoglossus semilaevis*). PLoS One.

[29] Tong JG, Sun XW (2015). Genetic and genomic analyses for economically important traits and their applications in molecular breeding of cultured fish. Sci China Life Sci.

[30] Zhou J, Wu Q, Wang Z, Ye Y (2004). Molecular phylogeny of three subspecies of common carp *Cyprinus carpio*, based on sequence analysis of cytochrome *b* and control region of mtDNA. J Zool Syst Evol Res.

[31] Zhao L, Zhang Y, Ji P, Zhang X, Zhao Z, Hou G (2013). A dense genetic linkage map for common carp and its integration with a BAC-based physical map. PLoS One.

[32] Ji P, Liu G, Xu J, Wang X, Li J, Zhao Z (2012). Characterization of common carp transcriptome: sequencing, *de novo* assembly, annotation and comparative genomics. PLoS One.

[33] Yan X, Ding L, Li Y, Zhang X, Liang Y, Sun X (2012). Identification and profiling of microRNAs from skeletal muscle of the common carp. PLoS One.

[34] Xu P, Zhang X, Wang X, Li J, Liu G, Kuang Y (2014). Genome sequence and genetic diversity of the common carp, *Cyprinus carpio*. Nat Genet.

[35] Peng W, Xu J, Zhang Y, Feng J, Dong C, Jiang L (2016). An ultra-high density linkage map and QTL mapping for sex and growth-related traits of common carp (*Cyprinus carpio*). Sci Rep.

[36] Laghari MY, Zhang Y, Lashari P, Zhang X, Xu P, Xin B (2013). Quantitative trait loci (QTL) associated with growth rate trait in common carp (*Cyprinus carpio*). Aquacult Int.

[37] Zhang Y, Wang S, Li J, Zhang X, Jiang L, Xu P (2013). Primary genome scan for complex body shape-related traits in the common carp *Cyprinus carpio*. J Fish Biol.

[38] Laghari MY, Lashari P, Zhang X, Xu P, Narejo NT, Liu Y (2014). Mapping QTLs for swimming ability related traits in *Cyprinus carpio* L. Mar Biotechnol.

[39] Van Ooijen JW (2006). JoinMap 4, software for the calculation of genetic linkage maps in experimental populations.

[40] Chakravarti A, Lasher LK, Reefer JE (1991). A maximum likelihood method for estimating genome length using genetic linkage data. Genetics.

[41] Fishman L, Kelly AJ, Morgan E, Willis JH (2001). A genetic map in the *Mimulus guttatus* species complex reveals transmission ratio distortion due to heterospecific interactions. Genetics.

[42] Hinegardner R, Rosen DE (1972). Cellular DNA content and the evolution of teleostean fishes. Am Nat.

[43] Maak S, Boettcher D, Tetens J, Swalve HH, Wimmers K, Thaller G (2010). Expression of microRNAs is not related to increased expression of ZDHHC9 in hind leg muscles of splay leg piglets. Mol Cell Probes.

[44] Boucher J, Masri B, Daviaud D, Gesta S, Guigné C, Mazzucotelli A (2005). Apelin, a newly identified adipokine up-regulated by insulin and obesity. Endocrinology.

[45] Genkai N, Homma J, Sano M, Tanaka R, Yamanaka R (2006). Increased expression of pituitary tumor-transforming gene (PTTG)-1 is correlated with poor prognosis in glioma patients. Oncol Rep.

[46] Mareco EA, de la Serrana DG, Johnston IA, Dal-Pai-Silva M (2015). Characterization of the transcriptome of fast and slow muscle myotomal fibres in the pacu (*Piaractus mesopotamicus*). BMC Genomics.

[47] Tian M, Li Y, Jing J, Mu C, Du H, Dou J (2015). Construction of a high-density genetic map and quantitative trait locus mapping in the sea cucumber *Apostichopus japonicus*. Sci Rep.

[48] Yu X, Zhou T, Li K, Li Y, Zhou M (1987). On the karyosystematics of cyprinid fishes and a summary of fish chromosome studies in China. Genetica.

[49] Saupe S, Bernard P, Laurent-Brun E, Derancourt J, Roizès G (1998). Construction of a human *Bcg*I DNA fragment library. Gene.

[50] Smith RM, Jacklin AJ, Marshall JJT, Sobott F, Halford SE (2013). Organization of the BcgI restriction–modification protein for the transfer of one methyl group to DNA. Nucleic Acids Res.

[51] The product information of *Bcg*I. https://www.neb.com/products/r0545-bcgi. Accessed 9 July 2017.

[52] Wang JT, Li JT, Zhang XF, Sun XW (2012). Transcriptome analysis reveals the time of the fourth round of genome duplication in common carp (*Cyprinus carpio*). BMC Genomics.

[53] Wang Y, Lu Y, Zhang Y, Ning Z, Li Y, Zhao Q (2015). The draft genome of the grass carp (Ctenopharyngodon idellus) provides insights into its evolution and vegetarian adaptation. Nat Genet.

[54] Xia JH, Liu F, Zhu ZY, Fu J, Feng J, Li J (2010). A consensus linkage map of the grass carp (*Ctenopharyngodon idella*) based on microsatellites and SNPs. BMC Genomics.

[55] Nakatani Y, Takeda H, Kohara Y, Morishita S (2007). Reconstruction of the vertebrate ancestral genome reveals dynamic genome reorganization in early vertebrates. Genome Res.

[56] Kasahara M, Naruse K, Sasaki S, Nakatani Y, Qu W, Ahsan B (2007). The medaka draft genome and insights into vertebrate genome evolution. Nature.

[57] Gjerde B, Gjedrem T (1984). Estimates of phenotypic and genetic parameters for carcass traits in Atlantic salmon and rainbow trout. Aquaculture.

[58] Luo W, Zeng C, Deng W, Robinson N, Wang W, Gao Z (2014). Genetic parameter estimates for growth-related traits of blunt snout bream (*Megalobrama amblycephala*) using microsatellite-based pedigree. Aquac Res.

[59] Taheri S, Murphy K, Cohen M, Sujkovic E, Kennedy A, Dhillo W (2002). The effects of centrally administered apelin-13 on food intake, water intake and pituitary hormone release in rats. Biochem Bioph Res Co.

[60] Castan-Laurell I, Dray C, Knauf C, Kunduzova O, Valet P (2012). Apelin, a promising target for type 2 diabetes treatment?. Trends Endocrin Met.

[61] Xu YX, Zhu ZY, Lo LC, Wang CM, Lin G, Feng F (2006). Characterization of two parvalbumin genes and their association with growth traits in Asian seabass (*Lates calcarifer*). Anim Genet.

[62] Nagy A, Csanyi V (1984). A new breeding system using gynogenesis and sex-reversal for fast inbreeding in carp. Theor Appl Genet.

[63] Devlin RH, Nagahama Y (2002). Sex determination and sex differentiation in fish: an overview of genetic, physiological, and environmental influences. Aquaculture.

[64] Takehana Y, Naruse K, Hamaguchi S, Sakaizumi M (2007). Evolution of ZZ/ZW and XX/XY sex-determination systems in the closely related medaka species, *Oryzias hubbsi* and *O. dancena*. Chromosoma.

[65] Chen J, Wang Y, Yue Y, Xia X, Du Q, Chang Z (2009). A novel male-specific DNA sequence in the common carp, *Cyprinus carpio*. Mol Cell Probes.

[66] Komen J, Yamashita M, Nagahama Y (1992). Testicular development induced by a recessive mutation during gonadal differentiation of female common carp (*Cyprinus carpio*, L.). Develop Growth Differ.

[67] Bradley KM, Breyer JP, Melville DB, Broman KW, Knapik EW, Smith JR. An SNP-based linkage map for zebrafish reveals sex determination loci. G3. 2011;1:3–9. 10.1534/g3.111.000190 PMC3178105 21949597

[68] Anderson JL, Marí AR, Braasch I, Amores A, Hohenlohe P, Batzel P (2012). Multiple sex-associated regions and a putative sex chromosome in zebrafish revealed by RAD mapping and population genomics. PLoS One.

[69] Eshel O, Shirak A, Weller JI, Slossman T, Hulata G, Cnaani A (2011). Fine-mapping of a locus on linkage group 23 for sex determination in Nile tilapia (*Oreochromis niloticus*). Anim Genet.

[70] Eisbrenner WD, Botwright N, Cook M, Davidson EA, Dominik S, Elliott NG (2014). Evidence for multiple sex-determining loci in Tasmanian Atlantic salmon (*Salmo salar*). Heredity.

[71] Taggart JB, Hynes RA, Prodöuhl PA, Ferguson A (1992). A simplified protocol for routine total DNA isolation from salmonid fishes. J Fish Biol.

[72] Fu X, Dou J, Mao J, Su H, Jiao W, Zhang L (2013). RADtyping: an integrated package for accurate de novo codominant and dominant RAD genotyping in mapping populations. PLoS One.

[73] Wang D, Liao X, Cheng L, Yu X, Tong J (2007). Development of novel EST-SSR markers in common carp by data mining from public EST sequences. Aquaculture.

[74] Ji P, Zhang Y, Li C, Zhao Z, Wang J, Li J (2012). High throughput mining and characterization of microsatellites from common carp genome. Int J Mol Sci.

[75] Voorrips RE (2002). MapChart: software for the graphical presentation of linkage maps and QTLs. J Hered.

[76] Langmead B (2010). Aligning short sequencing reads with bowtie. Curr Protoc Bioinformatics.

[77] Krzywinski M, Schein J, Birol I, Connors J, Gascoyne R, Horsman D (2009). Circos: an information aesthetic for comparative genomics. Genome Res.

[78] Van Ooijen JW (2009). MapQTL 6, Sofware for the mapping of quantitative trait loci in experimental populations of diploid species.

