# Supplementary material for: A high-resolution genetic linkage map and QTL fine mapping for growth-related traits and sex in the Yangtze River common carp (Cyprinus carpio haematopterus)
Source: BMC Genomics. 2018 Apr 2;19:230. doi: 10.1186/s12864-018-4613-1 (PMC5879560; doi:10.1186/s12864-018-4613-1)
Supplement: Supplementary file 2 — Figure S1. Genomic synteny visualized using Oxford grids between linkage groups of C. c. haematopterus and LGs of C. c. carpio. (PDF 1100 kb) [file 12864_2018_4613_MOESM2_ESM.pdf]

Cyprinus carpio carpio LGs

Cyprinus carpio haematopterus LGs

	1	2	3	4	5	6	7	8	9	10	11	12	13	14	15	16	17	18	19	20	21	22	23	24	25	26	27	28	29	30	31	32	33	34	35	36	37	38	39	40	41	42	43	44	45	46	47	48	49	50
1	12	14	1		2	1	1	1	3	2	1	2	3	1	2	1	1						1			2	2	1		1	2	3	2		1	1	1	2		1			1					2		1
2	5	33			2		4	1		1			1			1				1			1			3	3				2	2	2		4		1	1		2	2	1	2			1			1	1
3		1	22	4	1	1		1	1	1	3		1		1	2	1	1		1	2		1					1	1		1	2		3			2	1		1				2			1	5		
4			12	15				1	2	4				2		2		1								2		3	2	1	1	3	1	6	1					2		2		2			2	3	1	
5		1	2		18	18	2		1	3			1		1	2		3		1	2		1				5	2	3			1	3	2	2	2		1	1	2	1			1	5		2			
6		4	3		4	18	2	1	2		2		2	1	3	1		2		1	1	2	2							3	1	3	4	1				2	1				1	2	3			1	2	1
7	3	2				1	21	17		1	1	1		3	1	1	2	2	1		2	1						1	4	1	1		3	2	4	3			1		1	1		3	2			1		
8					2		10	26	1	2			1	1				1		1			1	1						3	1	1	3					2		5	2	1	1	1	1		4	2		
9		1	2				1	1	12	8		1				1		2	1			1		1		3		1			1		1	1	1		1						1					5		
10	3		1	1			2		16	23	1	2	1	1			2	2								1		2	4		4		2	2	4			4		1	1	4			2				1	1
11								2	5	3	25	4		1		3								3				2				2	2			1		1					1				1	1	1	1
12	1		1		1			1	1	1	10	14	1	1		1		1					1	1				1			3	3	1		4			1	2	1	1		2	1		1		2		
13	3	1	1		1	3	1	1	3		6		32	14		1		2			1		1	1	1	3	1	5	1	3		4	2	2	2	2		3	1	6	2	1	1	1	1			5		2
14	1		3	1		3		1			1		11	9	1		2	1								1	1	1		2	2	1	2		1	4	1			4	2	2	2	1		1	1	2	1	
15	2		3			1	2		6	5	2				17	13	1							1		3		1			2		2	3	3	2		3	1	4	1	1	1		2			1	1	
16	1		2	2			2	2	2	1	1	2			5	19		2								2		2			2	1			1	2	1	2	1		1		1	1			2	1		1
17		1	2		1	1		1		3			2			2	26	7	1	1			2	1		1					1			1	2	1	1			1	1		1	3		1				
18	2	1	1								2	1	2	1		2	3	14		1			1			1				1	1			2	1									1			1	5		1
19	1					6	1		2			1						1	9	7			1	1						1	1	1	5			1	1	2	2	1		3								2
20	1	2	3	2	1			3	1		2	4	1		1			1	3	15		1				2														1	1						1	1	1	
21	1	1	1		2	1		1	1			2	1			1	2				16	3		1		3		3		3	1	1		1	1		1			1	1		2	1	2	2		2		2
22					2	2		1	4		1					3	1	2			11	9						1		1	1		1		2	1								1				1		2
23	1				1				1	2	1			1				2			1		19	8		3	2				1	1	1		6	1			3	2	1		1	2			3	3		
24		1	2		1			1			2			1		1		3					2	8	1						2	1				2		2			2		1	1	2		2			
25			1		1		2			1	3			1						4		1		1	12	8		1	1		1	2	1	1	2		4	3		3		1				1	1			
26						1			1	1	1	1		3	1	2	1	1		1			1	1	4	9	1	1	1	2	2				3		3	4		1	3		1	1	1	1			1	
27						1		2	1	3	3		1	1			1	1	2	3	1		2			1	26	23	1			3	3	2	1	1	2			1	1	2	3	1			2	1		
28	4		1		2		2		2	1	3		3	1			2			1	1		2	1		1	6	19			1		2	3	2	2	1	4	1	1			1			2	1	2		
29					1					3	2		2										3	1				1	25	4			1	1				2		3			1	1		1		2		
30		4		1			1	1	3	1	2		3	3		1	2	1	2	1	1					2			14	29	1		1				2	1		3	1	1	2	1			1			
31			1					3	1	2	2		3	2				1	1		1		2			2	1		1	1	16	12			2	1		3				1			2			2		
32						1		3		1		3	3			1	1		1				1			3		2		3	13	38	1	1	1	1	1					1	1							
33	1	2		1	1			4			1	1						5					4			1		2	1	1	1	1	30	16	2	1		1	1							2	2	1		
34	5	1		1		2	1	2	1				1							2						2		4	2		2		4	16	2			2		1				1			1			
35		1			2	1	3			3				3		1		1			2		1		1	2		1	3		2	1	4	1	34	8	2	3	1	3	1	1	1					1		
36	1			3		1	1			1	1							1					1				2			1	1		1		12	36			1		1				2		1	1		
37	1		2	2	1	1			1	2		3	1			1	1			1							1	4						1			13	22		2					2			1		1
38	2	1					1				3	1	2		2	2		2				3					1	3		7	1	1	7	1	1		2	18		3		1		2	1		1		2	
39	2		2						1		1	2	1	4	2		1	1		1	1		2					1	3	1	2	1				2			18	5			1			2	3	1		1
40				1		2							1					1					2		1					3		3	2		1	1			7	23	1	1			1		1			
41	1				1		1		1						1	1		2				1		2				1		1	2	1	1	1	1	2		1			15	12	4		6	2			1	
42			2	1				1	1	2			1				1		2								1			1		1		1							4	14					2		4	
43	1				1	3				1	1	2	1	1							1		2		1				2	2								2				1	11	6				1		
44				1		1		4		1			2		1			3					1		1	3	2	1	1		3	1		1		1		1	1	1	1		6	7			1			1
45	1				1	1		1		1	1		2				1						3			1			1	1				1		1	1	1	2	1					13	9	1		3	
46	1		1	1	1				1	1	2		2		2			1				1				2	2	1	1	1	1		1			2		1	2	1				1	10	8	3	1	1	1
47	1		1			1	1					2		1		1							3							3		2	3	1		2		1		1	3						18	8		
48	3	1			1						1	1		1						1						1			1	3				1		1		1	1	1							12	27		2
49	1	1	1			2			2	3	3			2				2		1	1	1	2					1			1	1	1		4						1		1				3	1	12	2
50	2		1		2					2	1	3		1		1	2	3		1			1				1	1		1			1		2	1			1		1				2			1	9	15
