# Supplementary material for: A high-resolution genetic linkage map and QTL fine mapping for growth-related traits and sex in the Yangtze River common carp (Cyprinus carpio haematopterus)
Source: BMC Genomics. 2018 Apr 2;19:230. doi: 10.1186/s12864-018-4613-1 (PMC5879560; doi:10.1186/s12864-018-4613-1)
Supplement: Supplementary file 3 — Figure S2. Genomic synteny visualized using Oxford grids between linkage groups of C. c. haematopterus and chromosomes Danio rerio. (PDF 32 kb) [file 12864_2018_4613_MOESM3_ESM.pdf]

Danio rerio chromosomes

Cyprinus carpio haematopterus LGs

	Chr1	Chr2	Chr3	Chr4	Chr5	Chr6	Chr7	Chr8	Chr9	Chr10	Chr11	Chr12	Chr13	Chr14	Chr15	Chr16	Chr17	Chr18	Chr19	Chr20	Chr21	Chr22	Chr23	Chr24	Chr25
1	14																								
2	2						1									1			1						
3		11									1														
4		5																							
5			18				1																		
6			5											1											
7				11				1																	
8				4										1											
9			1		5																				
10	1				19																		1		
11						12																			
12				1		10													1						
13						1	19																		
14							16		1																
15								14																	
16								13																	
17				1					9																
18									7																
19										3			1												
20					1					5								1							
21											7														
22											7														
23												8													
24												5													
25													7												
26													8												
27														11									1		
28														11											
29															8										
30															14										
31																10									
32																10									
33																	18	1							
34		1															19								
35																	1	13							
36																		12						1	
37																			9						
38																			9						
39											1									9	1				
40																				7					
41																1					10				
42									1												5				
43																						2			
44																			1			7			
45																							5		
46					1														1			1	9		
47																								12	
48																			1					10	
49																1									4
50						1																			6
