# Supplementary material for: A high-resolution genetic linkage map and QTL fine mapping for growth-related traits and sex in the Yangtze River common carp (Cyprinus carpio haematopterus)
Source: BMC Genomics. 2018 Apr 2;19:230. doi: 10.1186/s12864-018-4613-1 (PMC5879560; doi:10.1186/s12864-018-4613-1)
Supplement: Supplementary file 4 — Figure S3. Genomic synteny visualized using Oxford grids between linkage groups of C. c. haematopterus and LGs of Ctenopharyngodon idellus. (PDF 31 kb) [file 12864_2018_4613_MOESM4_ESM.pdf]

Ctenopharyngodon idellus LGs

Cyprinus carpio haematopterus LGs

	8	22	2	11	17	18	1	21	16	24	10	9	5	13	15	12	23	7	4	14	3	6	19	20
1	16																						1	
2	5																							
3		18									1													
4		13												1										
5			19							1				5										
6			9											1			1							
7				11										1				1						
8				9										1										
9					9									1										
10					18																			
11						22																		
12						18													1					
13				1			22																	
14			1				18					1												
15								14																
16								19																
17									12															
18									8															
19										7														
20										4														
21											13									1				
22											10									2				
23						1						16												
24	1											5												
25													15							1				
26													12			1								
27										1				23										
28														19										
29										1					4				1					
30		2													1						1			
31																10			1					
32																15								
33																	12			1				
34																	19							
35													1			1		13						
36																		12			1			
37																			14					
38																			11					
39											1									10				
40																				13				
41																1					17			
42																					12			
43										2														
44								1		10														
45																						15		
46																						13		
47										1													15	
48					1														1				12	
49																1								4
50								1																4
