# Supplementary figures and images for: A high-resolution genetic linkage map and QTL fine mapping for growth-related traits and sex in the Yangtze River common carp (Cyprinus carpio haematopterus)
Source: BMC Genomics. 2018 Apr 2;19:230. doi: 10.1186/s12864-018-4613-1 (PMC5879560; doi:10.1186/s12864-018-4613-1)

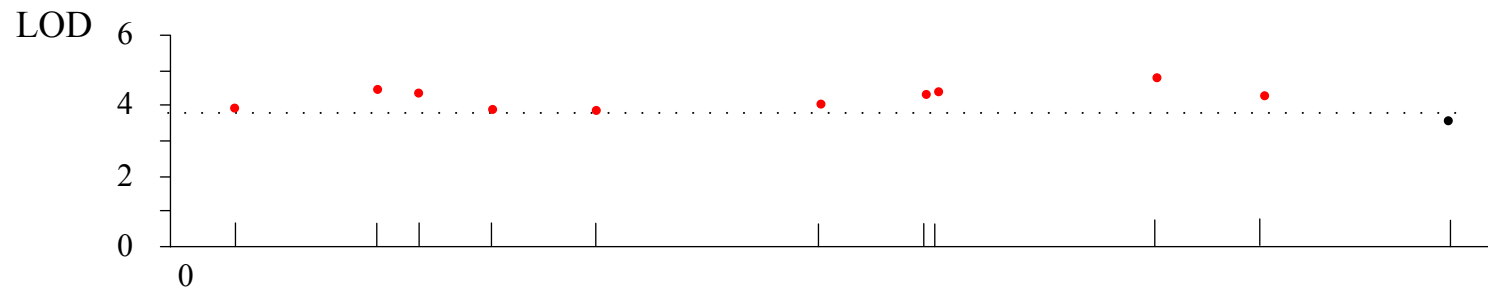

*C. Idellus*

Scaffold CI01000034

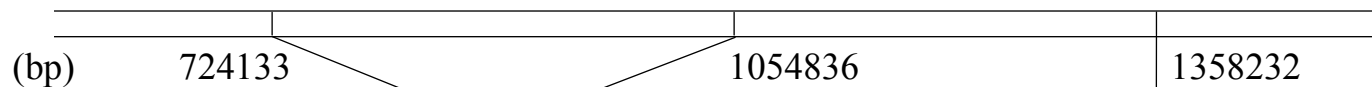

*C. c. haematopterus*

LG5

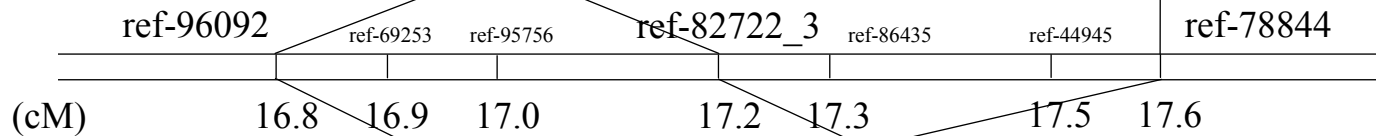

*D. rerio*

Chr3

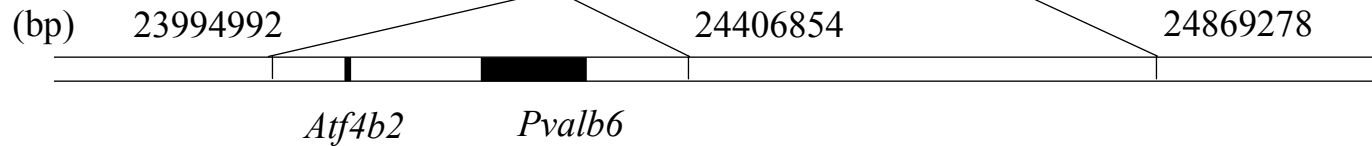

Supplement: Supplementary file 6 — Figure S4. The QTL region for head length on LG5 of C. c. haematopterus and its homologous region in genomes of Danio rerio and Ctenopharyngodon idellus. (PDF 135 kb) [file 12864_2018_4613_MOESM6_ESM.pdf]
